# Distinct Molecular Mechanisms of Altered HLA Class II Expression in Malignant Melanoma

**DOI:** 10.3390/cancers13153907

**Published:** 2021-08-03

**Authors:** Stefanie Meyer, Diana Handke, Anja Mueller, Katharina Biehl, Markus Kreuz, Jürgen Bukur, Ulrike Koehl, Maria-Filothei Lazaridou, Mark Berneburg, André Steven, Chiara Massa, Barbara Seliger

**Affiliations:** 1 Department of Dermatology, University Hospital of Regensburg, Franz-Josef-Strauss-Allee 11, 93053 Regensburg, Germany; smeyer@haut-schoen.de (S.M.); mark.berneburg@ukr.de (M.B.); 2 Institute of Medical Immunology, Martin Luther University Halle-Wittenberg, Magdeburger Str. 2, 06112 Halle (Saale), Germany; diana.handke@uk-halle.de (D.H.); anja.mueller@uk-halle.de (A.M.); katharina.biehl@uk-halle.de (K.B.); juergen.bukur@verwaltung.uni-halle.de (J.B.); marifili.lazaridou@uk-halle.de (M.-F.L.); andre.steven@uk-halle.de (A.S.); chiara.massa@medizin.uni-halle.de (C.M.); 3 Fraunhofer Institute for Cell Therapy and Immunology, Perlickstr. 1, 04103 Leipzig, Germany; markus.kreuz@izi.fraunhofer.de (M.K.); ulrike.koehl@izi.fraunhofer.de (U.K.)

**Keywords:** HLA class II, CIITA, IFN, methylation, signal transduction

## Abstract

**Simple Summary:**

There exists limited knowledge about the underlying molecular processes controlling the expression of HLA class II APM components and their prognostic significance in melanoma. Therefore, this study analyzed the basal and regulated expression of HLA class II antigens and components in melanoma cell lines and patients’ lesions in conjunction to T-cell infiltration. The heterogeneous constitutive HLA class II APM expression was caused by distinct molecular mechanisms and was partially linked to immune cell infiltration and clinical parameters. These results contribute not only to a better understanding of the regulation of HLA class II expression in melanoma, but might have an impact on the design of novel (immuno)therapies for the treatment of this disease.

**Abstract:**

Background: The human leukocyte antigen (HLA) class II molecules are constitutively expressed in some melanoma, but the underlying molecular mechanisms have not yet been characterized. Methods: The expression of HLA class II antigen processing machinery (APM) components was determined in melanoma samples by qPCR, Western blot, flow cytometry and immunohistochemistry. Immunohistochemical and TCGA datasets were used for correlation of HLA class II expression to tumor grading, T-cell infiltration and patients’ survival. Results: The heterogeneous HLA class II expression in melanoma samples allowed us to characterize four distinct phenotypes. Phenotype I totally lacks constitutive HLA class II surface expression, which is inducible by interferon-gamma (IFN-γ); phenotype II expresses low basal surface HLA class II that is further upregulated by IFN-γ; phenotype III lacks constitutive and IFN-γ controlled HLA class II expression, but could be induced by epigenetic drugs; and in phenotype IV, lack of HLA class II expression is not recovered by any drug tested. High levels of HLA class II APM component expression were associated with an increased intra-tumoral CD4+ T-cell density and increased patients’ survival. Conclusions: The heterogeneous basal expression of HLA class II antigens and/or APM components in melanoma cells is caused by distinct molecular mechanisms and has clinical relevance.

## 1. Introduction

The implementation of high-throughput technologies led to the identification of a large series of mutations, which appeared to be involved in the development, maintenance and progression of malignant melanoma (MM), but might also serve as suitable targets for T-cell-based immunotherapies, due to the creation of neo-antigens [1,2,3]. Despite that tumor-associated antigens (TAA) can be recognized by CD8+ cytotoxic T lymphocytes (CTL) in the context of HLA class I antigens, T-cell-based immunotherapies of melanoma might exhibit a lower efficacy than expected [4], and patients often develop resistances to these treatments [5]. This impaired response of MM patients is often associated with a downregulation or loss of HLA class I antigens and/or components of the HLA class I antigen-processing machinery (APM), leading to evasion from immune surveillance [6,7,8], disease progression and/or poor patients’ outcome [9,10], but their expression could be frequently upregulated by interferon (IFN)-α and IFN-γ [11,12]. There exists increasing evidence that HLA class II molecules encoded by HLA-DP, -DQ and -DR are also important for mounting an anti-tumoral immune responses, which can influence the prognosis of patients with various solid tumors and the efficacy of immunotherapies [13,14,15,16,17]. Recently, HLA class II surface expression has been shown to predict responses to anti-PD-1, but not to anti-CTLA-4 immunotherapy [18], suggesting its use as a potential biomarker for the responses prediction to specific immune checkpoint inhibitors (iCPIs) [14].

HLA class II surface molecules present foreign antigens to CD4+ T cells in order to initiate, control and/or maintain adaptive immune responses [19,20]. The HLA class II APM is complex and involves a number of components including the chaperones HLA-DM and HLA-DO. The expression of HLA class II antigens is tightly controlled by various transcription factors (TF), which are known to bind to highly conserved proximal promoter sequences of the HLA class II molecules [20,21,22]. The non-DNA-binding class II transactivator protein (CIITA) mediates the interaction between co-factors, chromatin remodeling factors and the general transcription machinery [23,24,25] and is central, but not sufficient, for the transcription of HLA class II antigens, which requires the presence of the enhanceosome complex. CIITA expression is transcriptionally regulated in a cell-type-specific manner, using different promoters [26]. Furthermore, promoter hypermethylation and histone acetylation can control HLA-DRα and CIITA transcription [27,28,29,30,31,32]. The expression of CIITA and selected HLA class II APM components could be reconstituted by the treatment with demethylating agents, histone deacetylase inhibitors (HDACi) and IFN-γ [20,33]. Furthermore, gene transfer of CIITA into tumor cells resulted in a stimulation of tumor specific CD4+ T cells in vivo associated with a long-lasting protective immunity [34], as well as an increased repertoire of tumor-associated HLA class II antigens [35].

Next to its physiologic expression on antigen presenting cells (APC), constitutive HLA class II expression was also detected in malignant cells. In freshly isolated primary and metastatic melanoma, 50–60% of tumor cells expressed HLA class II antigens [36]. In some tumor entities, HLA class II expression was associated with a favorable prognosis [37,38,39], while it correlated with a more aggressive phenotype and a higher risk of metastases in other cancers [40,41,42,43]. Concerning MM, there exist conflicting results regarding the role of HLA class II antigens in disease outcome and therapy response. Furthermore, a distinct expression pattern of HLA class II antigens was found during melanoma progression, suggesting a dynamic role of HLA class II function [44].

Based on these data, an increased knowledge concerning the underlying molecular processes controlling the expression of HLA class II APM components and their prognostic significance in MM is required. These might range from mutations in HLA class II regulatory genes [45] to transcriptional, posttranscriptional and epigenetic control [28,46,47,48,49,50,51]. Furthermore, the immune-cell repertoire might influence the HLA class II expression, since HLA-class-II-pathway component expression has been shown to be associated with B and T cell infiltration [52]. Therefore, this study analyzed the basal expression of HLA class II antigens and selected components of the HLA class II APM in MM cell lines and lesions, its regulation by IFN-γ and epigenetic drugs and its correlation to T-cell infiltration, in order to delineate the processes leading to the heterogeneous HLA class II expression in this disease. The clinical relevance of these results was demonstrated by correlation of the HLA class II APM expression and immune cell infiltration to tumor grading and to the patients’ survival by analysis of a melanoma dataset from The Cancer Genome Atlas (TCGA). 

## 2. Materials and Methods

### 2.1. Melanoma Cell Lines, Cell Culture and Treatment

The melanoma cell lines used in this study were either provided by Dr. Soldano Ferrone (Harvard University, Boston, MA, USA) or obtained from ESTDAB cell bank (now transferred to the European Collection of authenticated cell cultures, https://www.phe-culturecollections.org.uk/products/celllines/generalcell/browse.jsp, accessed on 12 May 2021, and their characteristics have been described elsewhere [53,54]. The melanocytes were purchased from Lonza (Pharma&Biotech, Basel, Switzerland) and cultured in melanocytes growth medium (MGM-4 Bullet Kit; Lonza Biosciences, Basel, Switzerland). All melanoma cell lines (*n* = 47) were maintained in RPMI1640 medium supplemented with 1% 100 mM L-glutamine, 10% fetal calf serum (FCS) and respective antibiotics. 

For determination of the IFN inducibility of HLA class II expression, melanoma cell lines were either left untreated or treated for 24 and/or 48 h with 400 U/mL recombinant IFN-α (Prospec, Rehovot, Israel) or IFN-γ (PAN Chemicals, Sofia, Bulgaria) respectively. For epigenetic studies, melanoma cell lines were daily treated with fresh medium containing the demethylating agent 5′-aza-2′-desoxycytidine (AZA; Sigma, Saint Louis, MO, USA; 1–10 µM) or the histone deacetylase inhibitors (HDACi) entinostat (ENT; Selleck Chemicals, Munich, Germany; 1–5 µM) or trichostatin A (TSA; 200 ng/mL; Sigma, Taufkirchen, Germany), respectively, for the indicated time points alone and/or in combination with IFN-γ.

### 2.2. qPCR Analysis

Total cellular RNA from melanoma cell lines and melanocytes was prepared and reverse transcribed into cDNA, as recently described [54]. Then qPCR was performed on a Rotorgene 6.000 system (Corbett Research, Sydney, Australia), employing the platinum SYBR Green qPCR Supermix-UPG (Invitrogen, Carlsbad, CA, USA) and respective primers listed in Table S1, using standard protocols, as recently described [55]. The mRNA levels were normalized to the expression of glyceratealdehyde-3-phosphate dehydrogenase (GAPDH). Data were analyzed with a comparative quantification mode of the Rotor gene 6.000 software version 1.7. The results were normalized to melanocytes. All qPCR analyses were performed with RNA from at least 3 independent experiments. The scoring of the mRNA expression levels was based on cycles and categorized as “1” (>26 cycles), “2” (22–26 cycles), “3” (18–21 cycles), “4” (14–17 cycles) and “5” (10–13 cycles). 

### 2.3. Monoclonal Antibodies

For flow cytometry and/or immunohistochemistry (IHC), following monoclonal antibodies (mAb) for staining of HLA class II APM components, we used anti-pan-HLA class II, anti-HLA-DR, anti-HLA-DP, anti-HLA-DQ, anti-CIITA, anti-HLA-DM, anti-HLA-DO and anti-Ii (Table S2). Staining with an anti-HLA class I mAb (Table S2) was performed to determine the IFN responsiveness. 

### 2.4. Flow Cytometry 

For flow cytometry, 5 × 10^5^ cells either left untreated or treated for the indicated time points with IFN-γ and/or DAC, TSA or ENT, respectively, were incubated with a fluorescence-labeled anti-human pan-HLA class II; anti-human HLA-DR, -DP and -DQ mAbs; or isotype controls for 1 h. After washing, HLA class II surface expression was measured on a NAVIOS flow cytometer (Beckman Coulter, Brea, CA, USA) and analyzed by using the Kaluza Software. The data were expressed as x-fold increase in mean fluorescence intensity (MFI) of the total population over the isotype control. A representative gating strategy is shown in Figure S1. The x-fold MFI of 1 was scored negative; MFI > 50 was scored high; and MFI was between 10 and 50, was scored low (<20% positive cells) or was medium (>20% positive cells). 

### 2.5. Analysis of CIITA Methylation Pattern 

For determination of the methylation status of the CIITA promoter, combined bisulfite restriction analysis (COBRA) and direct sequencing of bisulfite-treated DNA was performed, as recently described [54]. For COBRA, a nested PCR was performed with primers (Table S1B), and PCR products were digested with the restriction enzymes BstUI, Taq I, RsaI or Hpy188I (New England Biolabs, Frankfurt, Germany), recognizing CpG-specific sequences. The PCR products were then separated on 3% agarose gels. For determination of the methylation pattern, the degree of cleavage compared to the corresponding uncleaved control was categorized into different groups, as previously described [54].

### 2.6. Immunohistochemistry of Tissue Microarrays (TMAs)

The expression of HLA class II APM pathway components was determined in a melanoma-specific TMA consisting of 368 primary malignant melanoma lesions with available pT status for 362 samples, 39 metastases and 62 benign nevi, using conventional IHC [55,56,57]. Paraffin-embedded tissue blocks were stained with the respective primary antibodies, overnight, at 4 °C, followed by immunostaining with the UltraView Universal Alkaline Phosphatase Red Detection Kit (Ventana, Tucson, AZ, USA), as recently described [56]. Slides were counterstained with hematoxylin, dehydrated and mounted. The immune reactivity was defined according to the following scoring system: 0 = negative, 1 = 1–20%, 2 = 20–50%, 3 = 50–70% and 4 = 70–100%.

### 2.7. Statistical Analysis 

Statistical analysis was performed with SigmaPlot Version 11 (Inpixon HQ, Palo Alto, CA, USA) using the Student’s *t*-test; *p*-values of < 0.05 were considered as significant and are indicated in the figures. Experiments were performed 2 or 3 times. For contingency table analysis, two-sided chi-square and Fisher’s exact test were employed for statistical correlation between clinicopathological and immunohistochemical parameters of the tumor samples. The tumor stage was correlated to the expression of HLA class II APM pathway components, CD4+ and CD8+ T cell infiltration, using the statistic software RV.4.0.2. Kruskal–Wallis tests 11 were applied to compare the expression of individual components between different pT stages. Bonferroni corrections were applied to adjust for multiple testing, and adjusted values of < 0.05 were considered significant.

### 2.8. Bioinformatics Evaluation of Clinical Relevance

For the correlation of HLA class II APM component expression with overall survival (OS) of melanoma patients, the r2 database (https://hgserver1.amc.nl, accessed on 20 April 2021 with the TCGA melanoma Tumor Skin Cutaneous Melanoma (SKCM) study was used. A total of 470 samples from melanoma metastasis were separately included in the analysis. A *p*-value of < 0.05 was considered as significant. The following settings were chosen: Kaplan–Meyer by gene expression; cutoff modus, median; and follow-up time, 370 months. Inclusion criteria: number of samples in subset, *n* = 468; no subset selected.

## 3. Results

### 3.1. HLA Class II Expression in Melanoma Cells 

**Figure 1:**
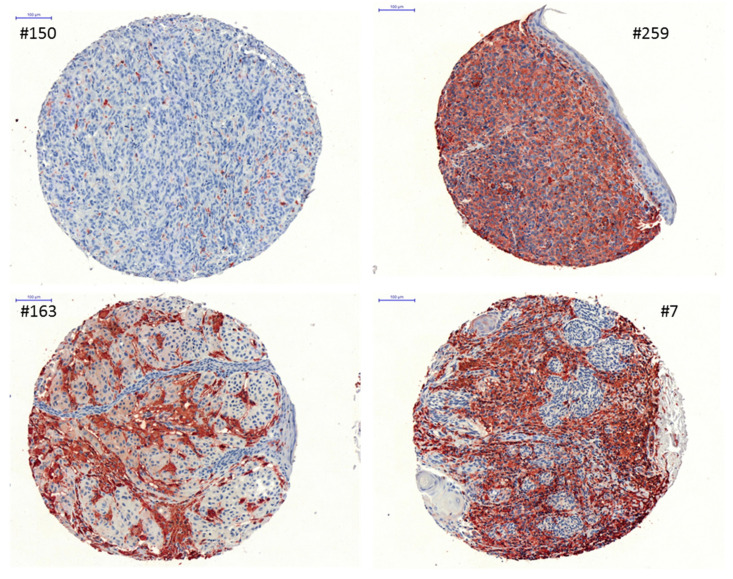
Representative immunohistochemical analysis of HLA class II antigens on melanoma lesions. IHC analysis was performed as described in Materials and Methods. Tissues were stained with the anti-HLA class II antibody LGII-612.14. Shown are representative stainings of a HLA class II negative (spot 150), HLA class II medium (spot 163) and HLA class II high (spots 7 and 259) expressing lesions. Scale bar: 100 µm.

Since the role of HLA class II antigens in melanoma is controversially discussed, a TMA consisting of 368 melanoma lesions was stained with an anti-pan-HLA class II-specific mAb antibody. Overall, 326/368 melanoma lesions gave informative IHC results, demonstrating a heterogeneous intratumoral staining. Representative stainings of melanoma lesions with a distinct HLA class II status (HLA class II^high^, HLA class II^medium^ and HLA class IIl^ow^) are shown in Figure 1. Overall, 126/326 melanoma lesions were defined as negative, 95/326 as low expressors, 68/326 as medium expressors and 37/324 as high expressors, with a staining mainly localized at the cell surface (Table 1). In contrast, no HLA class II expression was found in all benign nevi analyzed. 

The heterogeneous HLA class II antigen expression in situ was further confirmed in melanoma cell lines (*n* = 47), using melanocytes as a control. Moreover, 45% of melanoma cell lines (22/47) constitutively expressed HLA class II surface antigens, which varied from low/medium to high expression levels, while 25/47 melanoma cell lines lacked HLA class II surface expression. This led to the categorization of melanoma cell lines into negative, low/medium and high expressors (Table 1 and Table 2). 

### 3.2. Correlation of Heterogeneous HLA Class II Surface Antigen Expression with Altered APM Component Expression

Since the heterogeneous basal HLA class II surface expression detected in melanoma lesions and cell lines might be due to altered mRNA transcription, levels of the major HLA class II APM components were determined by qPCR. 

As shown in the heat map in Table 2 and summarized in Table 3, highly variable mRNA levels were detected for CIITA, HLA-DR, -DM and -DO; CLIP; the invariant chain (li); and cathepsin S in the melanoma cell lines analyzed, which were directly associated with HLA class II surface expression levels determined by flow cytometry (Table 2). Melanoma cell lines lacking HLA class II surface antigens expressed low-to-marginal transcript levels of some major HLA class II APM component, while high HLA class II expressors exerted high mRNA expression levels of most major HLA class II components analyzed (Table 2). Interestingly, the frequency of HLA class II component expression highly varied between the molecules (Table 3).

Furthermore, the immunohistochemical staining of the TMA demonstrated a heterogeneous expression pattern of HLA-DO and CIITA proteins in the melanoma lesions. Overall, 255/294 informative cases lacked HLA-DO expression, and 26/294 cases expressed low HLA-DO levels, while 13/294 cases analyzed expressed medium HLA-DO levels. In addition, a heterogeneous, but also a distinct expression pattern of CIITA was found between lymphocytes and tumor cells. In tumor cells, 284/314 cases lacked CIITA expression, 23/314 expressed low and 1/314 lesions medium levels of CIITA. In contrast, only 108/314 cases were negative for CIITA staining in immune cells, 139/314 cases showed a low, 55/314 cases a medium and 12/314 cases a high CIITA expression (Table 1). 

### 3.3. Distinct Responsiveness of Melanoma Cells to IFN-γ

**Figure 2:**
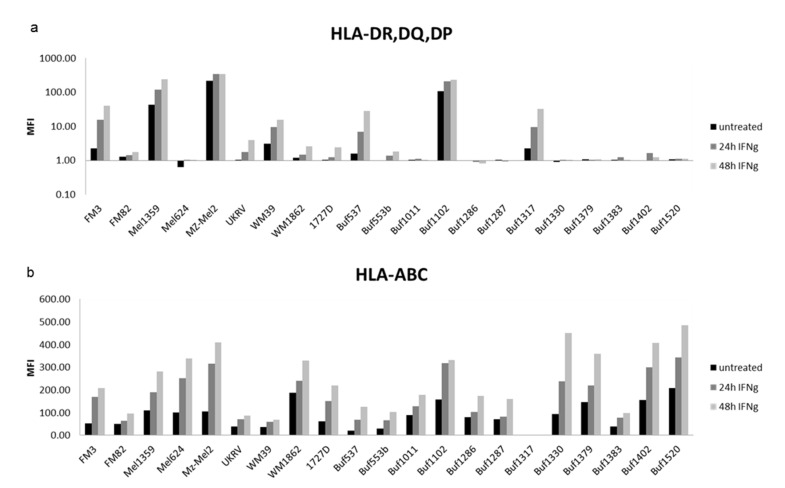
Heterogeneous basal and IFN-γ inducible surface expression of HLA class I and II antigens. Representative melanoma cells (*n* = 21) were left untreated or treated for 24 and 48 h with IFN-γ, before flow cytometric analysis of HLA class II surface expression was determined, as described in Material and Methods (**a**). Staining with a HLA-ABC antibody served as a control for IFN-γ responsiveness (**b**). The results are presented as x-fold in MFI over the isotype control.

**Figure 3:**
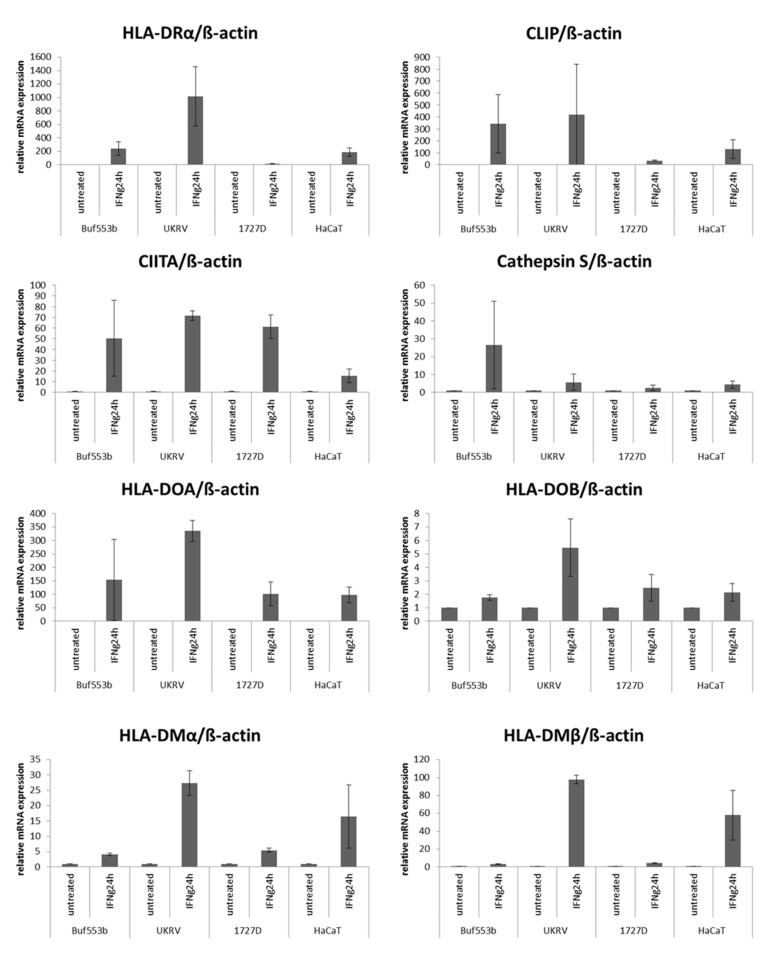
Link of IFN-γ-mediated upregulation of HLA class II surface expression with HLA class II APM components. The expression of HLA class II APM components of selected untreated and IFN-ƴ treated (24 h) melanoma cell lines was determined by qPCR. The data were normalized to β-actin expression, and relative mRNA expression was presented as bar charts by setting mRNA transcript levels of untreated cells to 1.

IFN-γ is a strong inducer of HLA class II APM expression in professional APC, but also in non-APC, including tumor cells [58]. Therefore, HLA-class-II-negative (16/25) and selected constitutively HLA-class-II-expressing (9/22) melanoma cell lines were treated with IFN-γ for 24 and 48 h prior to the analyses of HLA class II surface expression, using flow cytometry. Treatment of melanoma cells with IFN-α served as control. IFN-γ, but not IFN-α, treatment (Figure S2A) significantly upregulated HLA class II surface expression in 5/11 HLA class II-negative melanoma cells, but to a distinct extend regarding its kinetics and intensity (Figure 2a). As representatively shown for three selected melanoma cell lines and HaCat as a control, the upregulation of HLA class II mRNA (Figure 3) and surface expression (Figure 2a) by IFN-γ was associated with an increased expression of some HLA class II APM components, e.g., HLA-DO, HLA-DM, CIITA, CLIP and cathepsin S (Figure 3). It is noteworthy that both IFN-γ (Figure 2b) and IFN-α (Figure S2B) were able to upregulate HLA class I surface expression in these melanoma cell lines, suggesting a functional IFN-γ signaling pathway. 

### 3.4. Upregulation of HLA Class II Surface Expression upon Treatment of Melanoma Cells with Epigenetic Drugs

**Figure 4:**
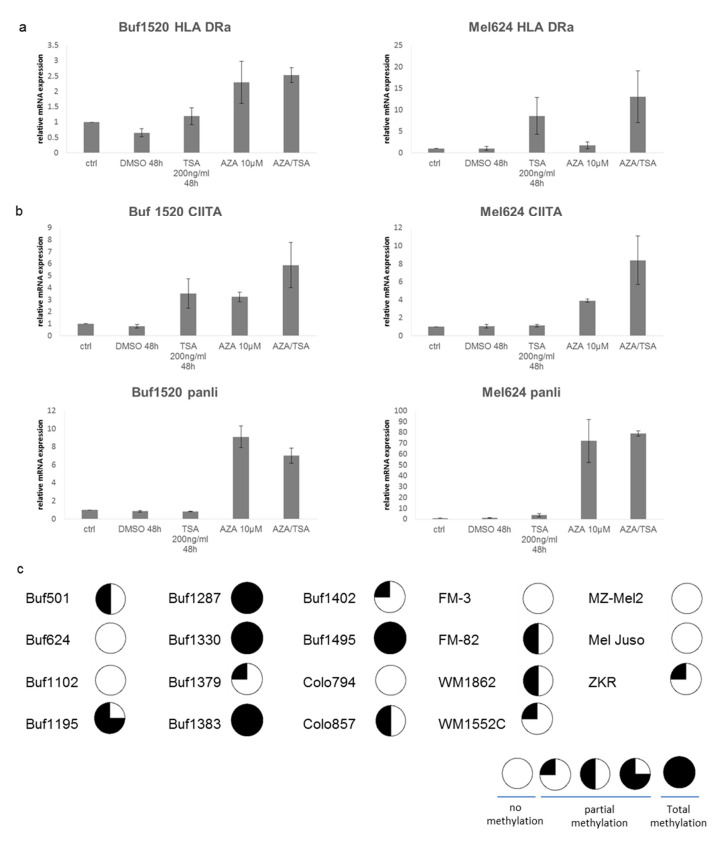
Epigenetic control of HLA class II APM components. Melanoma cells were left untreated or treated for 48 h with AZA (10 µM), TSA (200 ng/mL) and/or a combination of both, before mRNA expression of HLA-DR I (**a**) and HLA class II APM components (**b**) was determined by qPCR. The data are expressed as relative mRNA levels by setting untreated cells as 1. The data are shown as mean ± SE from two different experiments. (**c**) The methylation status of CIITA was analyzed in HLA class II negative melanoma cell lines, as described in Materials and Methods. The results are presented as total, partial (25–75%) and no methylation.

The lack of basal and IFN-ƴ inducible HLA class II expression was detected in 11/16 melanoma cell lines, which might be due to epigenetic silencing mediated by methylation or altered histone acetylation [47,59]. To study whether both processes are responsible for the lack of HLA class II surface expression, different melanoma cell lines were treated with AZA, HDACi or a combination of both for 5 days, followed by mRNA analysis of major HLA class II APM components or flow cytometric analysis of HLA class II surface antigens. Moreover, 2/11 HLA class-II-negative and IFN-γ resistant melanoma cell lines induced HLA class II mRNA expression (Figure 4a), but not HLA class II surface expression (data not shown), upon AZA treatment. In addition, AZA treatment had also no effect on HLA class II surface expression of constitutive HLA class II expressing melanoma cell lines. Treatment with TSA in combination with AZA had additive effects on the mRNA HLA class II expression (Figure 4a), suggesting that both methylation and histone acetylation are underlying molecular mechanisms impairing HLA class II mRNA expression in some melanoma cells [60]. The AZA- and TSA-mediated upregulation of HLA class II mRNA expression was accompanied by an increased expression of HLA class II APM components, but this significantly differed between the HLA class II APM components, as well as between the distinct cell lines analyzed. This is representatively shown for HLA-DR, CLIP and CIITA in Figure 4a,b. Similar results were obtained by using ENT as HDACi.

### 3.5. CIITA Expression as a Major Regulator of the HLA Class II Surface Expression

Since the HLA class II transactivator CIITA has been shown to be involved in chromatin remodeling [61] and the HLA class II surface antigens could be induced in some melanoma cell lines, it was analyzed whether the lack of HLA class II surface antigen is due to the methylation of the CIITA promoter, as already shown for gastric and colorectal cancer, for example [62]. Therefore, the CIITA methylation status of melanoma cells (19/47) was investigated by COBRA analysis [63], demonstrating a total or partial methylation (25–75%) of the CpG islands in the CIITA promoter. CIITA was frequently methylated in HLA class II^low/neg^. melanoma cells, which did not respond to IFN-γ despite a functional IFN-γ pathway (Figure 4c), but could be reverted by the AZA treatment. 

### 3.6. Correlation of HLA Class II APM Components and Immune Cell Infiltration with Clinical Relevance

**Figure 5:**
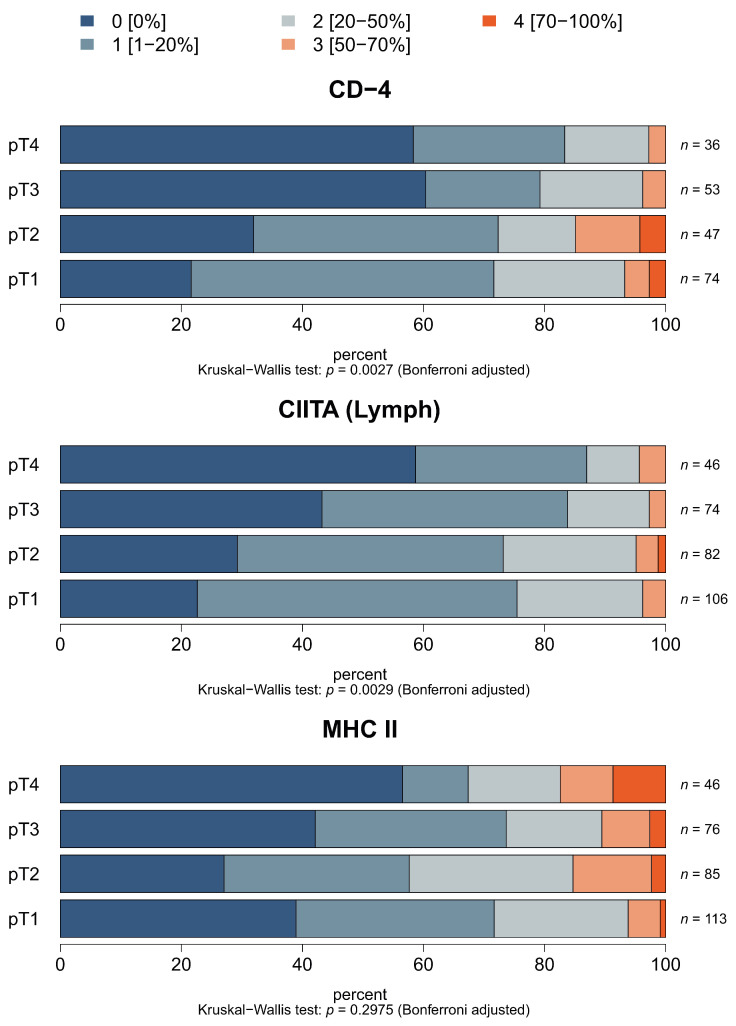
Correlation of HLA class II APM expression and immune cell infiltration with tumor staging. The staining of the TMA was performed as described in Materials and Methods. Expression of CIITA in lymphocytes and CD4^+^ T-cell infiltration correlated to pT stages of tumor samples.

In order to determine the clinical relevance of HLA class II expression, the basal HLA class II and/or CIITA expression of melanoma lesions, as well as the level of immune cell infiltration, was correlated to tumor staging. As shown in Figure 5, the distinct HLA class II expression was not correlated to tumor grading (*p* = 0.2975). In contrast, CIITA expression in lymphocytes, but not in tumor cells, correlated to tumor staging (*p* = 0.0029 and *p* = 1, respectively). The tumor stage was further associated with the frequency of CD4+ T-cell infiltration, which was the highest in pT1 melanoma and the lowest in pT4 tumors (*p* = 0.0027). However, no statistically significant correlation exists between tumor stage, IFN-γ and HLA-DO (Figure S3). In summary, tumor grading is correlated with CD4+ T-cell infiltration and CIITA expression. 

**Figure 6:**
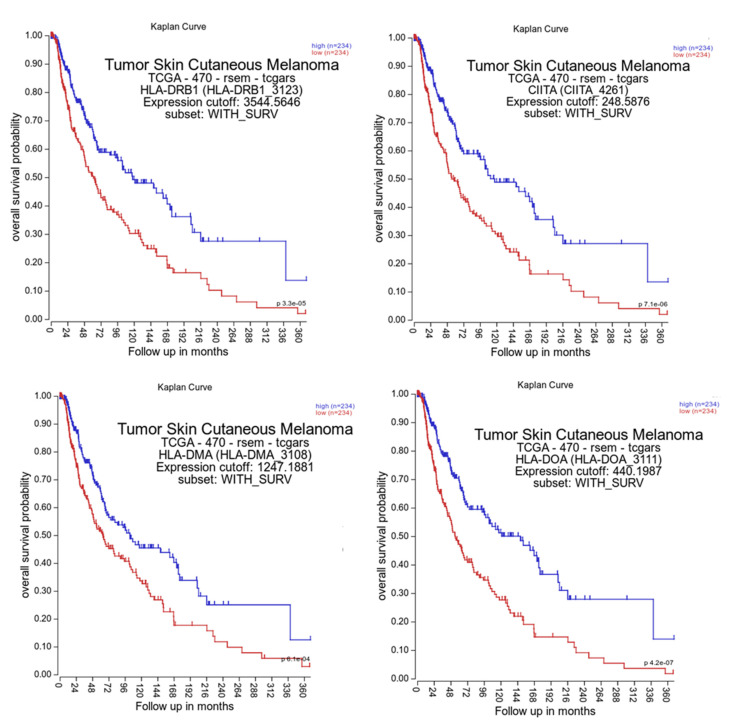
Correlation of HLA class II APM expression with the survival of melanoma patients, using TCGA data. For analysis of the OS of melanoma patients, the TCGA data were analyzed as described in Material and Methods.

Furthermore, for comparison of the prognostic relevance of HLA class II APM component expression, in silico analyses of TCGA data were performed by using datasets from 234^high^ and 234^low^ HLA class II APM component expressors. As shown in Figure 6, the HLA class II APM^high^ patient group has a significantly increased overall survival (OS) compared to patients with HLA class II APM ^low^ expression.

## 4. Discussion

Despite the fact that HLA class II expression is mainly found on APC, basal expression of HLA class II antigens has been also detected on distinct tumor types—particularly in hematopoietic malignancies, but also solid tumors—while others totally lack HLA class II expression. The constitutive HLA class II expression on tumor cells results in their recognition by tumor-antigen-specific CD4+ T cells, generating a Th1 response [64]. Since the role of HLA class II molecules in MM has not yet been characterized in detail, this study determined the frequency of basal HLA class II surface expression in a large cohort of melanoma lesions by staining different TMAs. These data were correlated to the immune cell infiltration and to tumor staging. In addition, a large number of melanoma cell lines (*n* = 47) were analyzed regarding their constitutive and inducible HLA class II surface antigen expression in order to determine the underlying molecular mechanisms of deficient HLA class II expression in MM. A distinct HLA class II surface expression pattern was found on both melanoma cell lines and melanoma lesions, with a relatively high frequency when compared to other solid tumors entities [15,65,66]. Low levels of HLA class II surface antigens in melanoma samples were associated with a high tumor grading, while high levels of HLA class II antigens were found in pT1 melanoma. These data suggested a clinical relevance of HLA class II antigen expression. In order to get further insights into the clinical impact of HLA class II expression the HLA class II staining pattern should have been correlated to the OS of patients. Unfortunately, survival data were not available for our TMA cohort. Therefore, we used TCGA data as surrogate analysis data, confirming the association of HLA class II and tumor staging and further extended these results to patients’ survival. 

It has been reported by various groups that the underlying mechanisms responsible for the highly variable HLA class II expression in tumors are broad, but they have been mainly characterized in hematological disorders [67]. These include different genomic alterations of HLA class II molecules, which have been identified in Non-Hodgkin lymphoma, such as deletions, mutations and chromosomal rearrangements, leading to an impaired HLA class II expression and resistance to IFN-γ treatment [68,69]. Loss of HLA class II expression and transcriptional silencing of HLA class II molecules frequently occur in leukemia relapses after human-stem-cell transplantation [70]. The deficient HLA class II expression could be reverted in some cases by IFN-γ treatment due to IFN-responsive elements in the promoters of some HLA class II APM components. In addition, epigenetic control including methylation and histone deacetylation is often involved in the lack of basal, as well as IFN-γ-induced expression of HLA class II surface antigens, and could be reverted by demethylating agents and HDAC [47,71]. Using melanoma cell lines as models, a classification of MM into four distinct phenotypes was established: phenotype I exhibits basal, but heterogeneous HLA class II expression. The phenotype II in MM lacks HLA class II surface expression, which is IFN-γ inducible and also accompanied by an upregulation of some major HLA class II APM components. Furthermore, IFN-γ not only influences HLA class II expression directly through the EnhA, ISRE and CRE elements, but also by upregulation of CIITA [72]. This appears to be associated with the level of T-cell infiltration due to IFN- γ secretion by immune cells. However, some melanoma cells lack not only basal, but also an IFN-γ mediated upregulation of HLA class II antigens despite a functional IFN-γ signaling pathway [73]. In phenotype III, the reduced or missing HLA class II expression is due to the epigenetic control, since treatment of melanoma cells with the demethylation agent AZA and/or the HDACi TSA results in an upregulation of HLA class II expression. Thus, DNA methylation or altered histone acetylation of HLA class II antigens plays an important role in the modulation of HLA class II APM component expression, which is also of clinical relevance. Combination of AZA and ENT significantly reduced tumor growth and increased patient-derived HLA class II expression in xenografts [74]. 

One key molecule involved in the regulation of basal HLA class II expression is CIITA, which is of pathologic relevance in rare, but severe immune disorders [75]. Furthermore, loss-of-function mutations in CIITA resulted in the lack of HLA class II expression, which was found in various tumor types [76], while a substitution of A to G in the 5′ flanking region of the CIITA promoter was associated with a higher expression [73]. Our data suggested that the methylation of the CIITA promoter in HLA class II negative, IFN-γ-resistant MM cell lines frequently occurred, but the methylation status between the MM cell lines analyzed was highly variable from total methylation to partial (25–75%) methylation. This is in line with reports demonstrating a frequent promoter methylation of CIITA in different cancer types that was associated with an impaired HLA class II expression and could not be reverted by IFN-γ, but by demethylating agents [77], as shown for ovarian cancer [78], diffuse large B-cell lymphoma [79] and breast cancer [80]. However, the lack of basal or downregulated CIITA expression might be due to other mechanisms, such as an upregulation of microRNAs (miRNAs) targeting the 3′UTR of CIITA [51]. 

The highly variable expression of HLA class II antigens and APM components is in line with TCGA RNA sequencing data, demonstrating an association of higher expression levels of HLA class II genes—in particular, of HLA-DP and -DR—with a better survival of melanoma patients [81]. The HLA class II expression of human tumor cells may contribute to an enhanced tumor immunity, since it can induce HLA class II-restricted CD4+ T-cell responses. Pathway analyses of melanoma cell lines expressing HLA class II antigens under basal or IFN-γ stimulated conditions demonstrated signatures for PD-L1 signaling, allograft rejection and T-cell-receptor signaling. Furthermore, HLA class II antigen expression was associated with an increased therapeutic response and improved patients’ outcome [14].

In previous publications, highly variable basal HLA class II expression levels were described in primary melanoma lesions and melanoma cell lines. These were also associated with an altered frequency of tumor infiltrating CD4+ and CD8+ T cells. However, controversial results regarding the role of HLA class II antigen expression for prognosis and OS of MM patients, and in particular for those treated with immunotherapies, exist [14,16,65,66,67]. Thus, there is an urgent need to further characterize the role of HLA class II antigens in the context of immune cell infiltration and determine the HLA class II expression in large cohorts of melanoma patients responding and non-responding to immunotherapy.

## 5. Conclusions

In this study, the expression of HLA class II antigen and APM component was evaluated on multiple melanoma cell lines, as well as in patients’ specimen. Different patterns of constitutive and inducible expression of HLA class II molecules were found, which also correlated with the patients´ clinical outcome. These results contribute not only to a better understanding of the regulation of HLA class II expression in melanoma, but might have an impact on the design of novel (immuno)therapies for the treatment of this disease.

## Figures and Tables

**Table 1:** Distinct protein expression pattern of HLA class II antigens and/or APM components in melanoma lesions and melanoma cell lines.

Samples	HLA-II APM	*n* ^1^	High	Medium	Low	Negative
Melanoma lesions	HLA class II	326	37 (11.33%)	68 (20.86%)	95 (29.14%)	126 (38.65%)
	HLA-DO	294	0	13 (4.42%)	26 (8.84%)	255 (86.73%)
	CIITA tumor	314	0	1 (0.32%)	23 (7.32%)	284 (90.45%)
	CIITA lymph.	314	12 (3.82%)	55 (17.52%)	139 (44.27%)	108 (34.39%)
Melanoma cell lines	HLA class II	47	4 (8.51%)	13 (27.66%)	5 (10.64%)	25 (53.19%)

^1^ Number of samples analyzed.

**Table 2:** Characterization of the HLA class II APM component expression in melanoma cell lines.

Cell Line	qPCR ^1^									Flow Cytometry ^2^	
	CIITA	HLA-DRa	HLA pan li	Cathepsin	HLA-DMa	HLA-DMb	HLA-DOA	HLA-DOB	CLIP	Classification	% Positive
melanocytes	2	2	3	3	3	2	1	1	2	neg.	
HaCAT	2	2	3	4	3	2	1	2	2	neg.	
BUF836	1	2	3	3	2	2	1	1	3	neg.	
Mel 624	1	1	1	2	3	2	1	1	1	neg.	
BUF1495	1	1	2	3	3	3	1	1	1	neg.	
BUF1286	1	1	2	3	3	3	2	1	1	neg.	
BUF1182	1	1	2	1	3	2	1	1	1	neg.	
BUF553b	1	1	1	3	3	2	1	1	1	neg.	
FM6	2	1	3	3	3	2	1	2	3	neg.	
BUF1330	1	1	2	3	3	3	1	1	1	neg.	
BUF501 ATC	1	1	2	3	3	2	1	1	1	neg.	
BUF1011	1	1	2	2	3	2	1	1	2	neg.	
BUF1195	1	1	1	1	2	1	1	1	1	neg.	
BUF1383	1	1	1	3	2	1	1	1	1	neg.	
FM81	1	1	1	3	3	3	2	2	1	neg.	
BUF1402	1	1	2	2	3	2	1	1	1	neg.	
BUF1287	2	3	5	3	3	2	2	1	3	neg.	
1727D	2	3	3	3	3	3	1	1	2	neg.	
SK Mel29 1	2	2	2	2	3	2	1	2	1	neg.	
BUF1280	1	1	1	1	2	1	1	1	1	neg.	
Brooks 86	1	1	1	3	3	2	1	1	1	neg.	
UKRV Mel14a	2	1	2	2	3	2	1	3	1	neg.	
BUF1379	1	1	2	2	3	2	1	1	1	neg.	
COLO 857	1	1	2	2	2	2	1	1	2	neg.	
NA-8	1	1	2	2	3	1	1	1	1	neg.	
BUF1520	1	1	3	2	2	2	1	1	3	neg.	
BUF624	1	2	2	3	3	2	1	1	2	neg.	
WM1862	1	2	3	3	3	2	1	2	2	low	11.9
MZ Mel3	2	4	3	2	3	3	1	1	3	low	13.3
M17	3	4	4	3	3	3	3	1	4	low	11.5
FM82	2	3	3	2	2	1	1	1	4	low	3.5
BUF1088	1	1	2	3	3	3	2	1	2	low	13.7
FM79	2	3	3	1	4	3	2	2	3	med.	23.3
BUF537	3	4	4	3	4	3	1	1	4	med.	27.4
FM28	3	4	4	3	4	3	2	2	3	med.	24.9
WM39	2	4	4	3	3	2	1	2	4	med.	all
FM3	4	5	5	2	4	4	4	3	5	med.	35.8
MKR	3	4	4	2	4	2	2	1	4	med.	58.4
WM1552c	1	2	1	3	3	3	2	2	1	med.	all
2058 Brooks	2	4	4	4	3	2	2	1	4	med.	all
BUF526	2	3	4	2	3	2	1	2	5	med.	all
BUF1317	2	3	4	2	3	2	1	2	4	med.	all
GR-M	3	4	4	3	3	2	1	2	4	med.	all
Mel1359	3	4	4	3	4	4	3	2	4	med.	all
Mel JUSO	4	5	5	1	4	4	4	2	5	med.	all
COLO794	3	4	5	3	4	4	2	2	4	high	all
MZMel2	4	5	5	2	4	4	4	3	5	high	all
ZKR	3	4	5	2	4	4	4	2	5	high	all
BUF1102	3	5	5	1	4	3	4	2	5	high	all

^1^ Heat map based on the takeoff: “1” is after 26 cycles, “2” between 22 and 26, “3” between 18 and 21, “4” between 14 and 17, and “5” between 10 and 13; ^2^ antibody staining.

**Table 3:** Heterogeneous mRNA levels of HLA class II APM components in melanoma cell lines.

HLA Component	Melanoma Cells		
	High	Medium	Negative
CIITA	3	21	23
CLIP	16	13	18
pan-li	16	23	8
HLA-DRa	15	11	21
HLA-DOA	5	12	30
HLA-DOB	0	19	28
cathepsin S	1	40	6
HLA-DMa	11	36	0
HLA-DMb	6	36	5

## Data Availability

The data and all materials are available upon request.

## Supplementary Materials

The following are available online at https://www.mdpi.com/article/10.3390/cancers13153907/s1. Figure S1: Gating strategy for melanoma cell lines. Figure S2: Lack of IFN-α inducibility of HLA class II, but not of HLA class I surface expression. Figure S3: Correlation of the expression of IFN-γ, CD8, CIITA (tu) and HLA-DOB to pT stages of melanoma samples and/or immune cells. Table S1: Primers used for PCR analysis. Table S2: Antibodies used for flow cytometric analysis of protein expression.
