# Supplementary material for: Distinct Molecular Mechanisms of Altered HLA Class II Expression in Malignant Melanoma
Source: Cancers (Basel). 2021 Aug 3;13(15):3907. doi: 10.3390/cancers13153907 (PMC8345549; doi:10.3390/cancers13153907)
Supplement: Supplementary file 1 [file cancers-13-03907-s001.zip › cancers-1320668-SI.pdf]

Supplementary Material: Distinct Molecular Mechanisms of Altered HLA Class II Expression in Malignant Melanoma

Stefanie Meyer, Diana Handke, Anja Müller, Katharina Biehl, Markus Kreuz, Jürgen Bukur, Ulrike Koehl, Maria-Filothei Lazaridou, Mark Berneburg, André Steven, Chiara Massa, Barbara Seliger

Table S1. Primers used for PCR analysis; Primers for the expression analysis of MHC class II APM components.

**A. Primer used for qPCR analysis.**			
**Transcription unit**	**Name**	**Sequence**	**Annealing Temperature/Accession number**
ACTB	ßactin fw	TCC TGT GGC ATC CAC GAA ACT	58 NM_001101.5
	ßactin rev	GAA GCA TTT GCG GTG GAC GAT	
GAPDH	huGAPDH fwd	GGA CTC ATG ACC ACA GTC CAT	60 NM_002046.7
	huGAPDH rev	CGG AAG GCC ATG CCA GTG AG	
ALAS1 tv1	ALAS1 RT fw	TGA GAC AGA TGC TAA TGG ATG C	60 NM_000688.6
	ALAS1 RT rev	CAC CGT AGG GTA ATT GAT TGC T	
HLA-DOA	hsHLA-DOA fwd rt	ACC CAT GAA TTT GAT GAG GAA C	60 NM_002119.4
	hsHLA-DOA rev rt	AGC CAG GTG ATA TTG ATC ACA G	
HLA-DOB	hsHLA-DOB fwd rt	GGC AAA GGC TGA CTG TTA CTT C	60 NM_002120.4
	hsHLA-DOB rev rt	GGT CCT CTC TGG GTA CAC TGT C	
CLIP tv1	hCLIP fwd qPCR	TAT CTC CAA CAA TGA GCA AC	60 NM 001025159.3
	hCLIP rev qPCR	TCT GTC ATG TTG CCA TAC TT	
Cathepsin S tv1	cathepsin S fw rt	CCT TAT GGC AGA GAA GAT GTC C	60 NM_004079.5
	cathepsin S rev rt	CAT AGC CAA CCA CAA GTA CAC C	
CIITA tv2	CIITA fw realtime	AGA AGT TCC TCG GAA GAC ACA G	60 NM_000246.4
	CIITA rev realtime	TGT TGT TCT GGG ACA GAT TGA G	
panli-HLA II tv1	pan li HLA II fw RT	TGG ACA AAC TGA CAG TCA CCT C	60 NM_001025159.3
	pan li HLA II rev RT	GTC CTC TGT CAT GTT GCC ATA C	
HLA-DR alpha	HLA-DR alpha fw rt	AAC TGA GGA CGT TTA CGA CTG C	60 NM_019111.5
	HLA-DR alpha rev rt	AAG ATG GTC CCA ATA ATG ATG C	
HLA-DM alpha	HLA-DM alpha fw rt	TTG ACA AAG AGT TCT GCG AGT G	60 NM_006120.4
	HLA-DM alpha rev rt	TGG GTG GGA AGA GAT TAC TGA C	
HLA-DM beta	HLA-DM beta fw rt	CCT CTC CCA TTT AGC CTT AAC C	60 NM002118.5
	HLA-DM beta rev rt	CAC TGC AGA CAC AGA AAC CTT C	
**B. Primers for the methylation analysis of CIITA by COBRA**			
Methylation	Name	Sequence	Annealing Temperature
hCIITA	hC2TA p4 cpG1 fwd	TTA GGT AGT TGG GAT GTT ATT TTT GAT A	60
	hC2TA p4 cpG1 rev	TCC TCC CAA ACT TAC TAT ATA ACC TTA AAC	60
	hC2TA p4 cpG2_3 fwd	AAG GTT ATA TAG TAA GTT TGG GAG GAT G	60
	hC2TA p4 cpG2_3 rev	TAT AAC CCA ACA AAA AAA ATA AAA CTC A	60

Table S2. Antibodies used for flow cytometric analysis of protein expression.

**A. Flow cytometry**		
**Antibody**	**Clone**	**Company**
Mouse IgG2a-FITC	7T4-1F5	Beckman Coulter (Krefeld, Germany)
HLA-ABC-FITC	B9.12.1	Beckman Coulter (Krefeld, Germany)
HLA-DR,DQ,DP-FITC	Tu39	Beckton Dickinson (Heidelberg, Germany)
HLA-DR-PE	G46-6	Beckton Dickinson (Heidelberg, Germany)
HLA-DQ-FITC	Tu169	Beckton Dickinson (Heidelberg, Germany)
HLA-DP-FITC	HI143	Immunostep (Salamanca, Spain)
mouse IgG2a-PE isotype control	G155-178	Beckton Dickinson (Heidelberg, Germany)
**B. Immunohistochemistry**		
**Antibody**	**Clone**	**Company**
CIITA	7-1H	Santa Cruz Biotechnology (Heidelberg, Germany)

HLA-DR	LGII-612.14	Soldano Ferrone (Boston, USA)
HLA-DO	polyclonal	Sigma-Aldrich (St. Louis, USA)

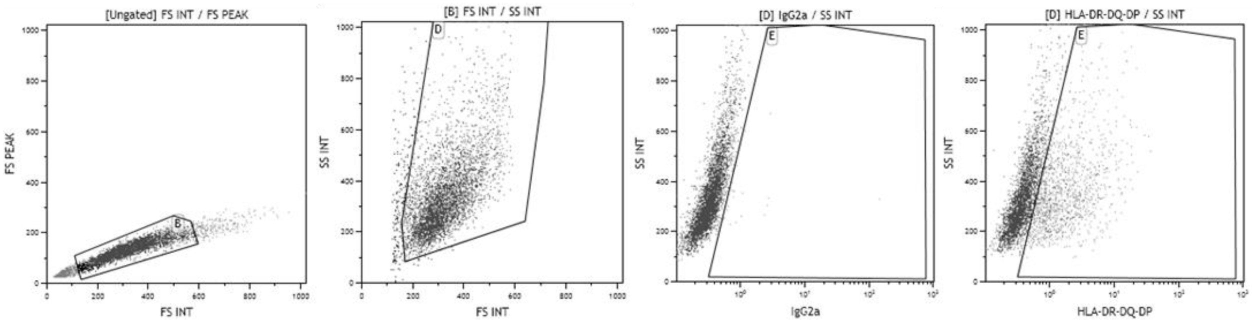

Figure S1. Gating strategy for the evaluation of HLA class II surface expression by melanoma cell lines. Melanoma cells were gated for single living cells based on the integral and peak value of the forward scatter (FS) and the combination of the forward and side scatter (SS), respectively. The percentage of cells expressing the antigen was obtained by setting a gate on the isotype control stained cells, whereas the mean fluorescence intensity (MFI) was calculated for the total population.

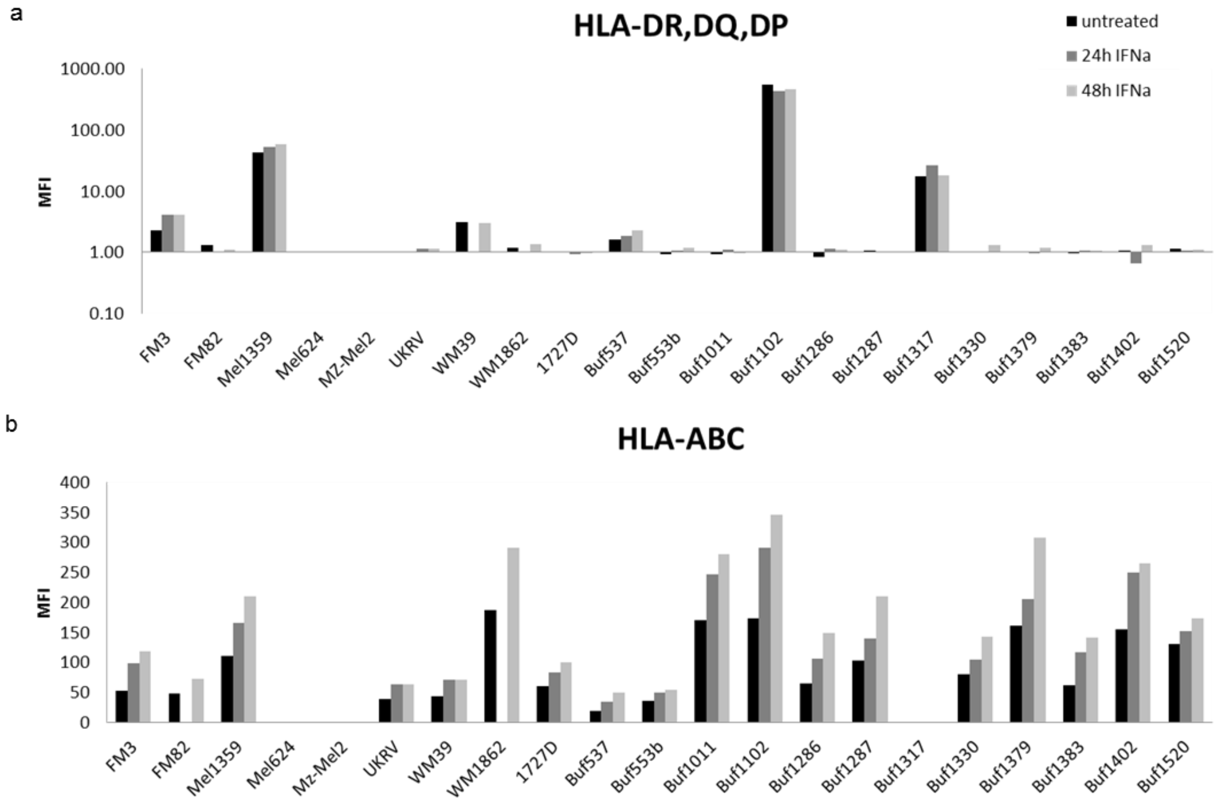

Figure S2. Lack of IFN-α inducibility of HLA class II, but not of HLA class I surface expression. Melanoma cells were left untreated or treated for 24 and 48 hrs with IFN-α, before flow cytometric analysis was performed using HLA class II (**a**) or HLA class I (**b**) specific antibodies, respectively. The results are presented as bar charts of relative MFI of HLA class II and class I setting the MFI of untreated cells as 1.

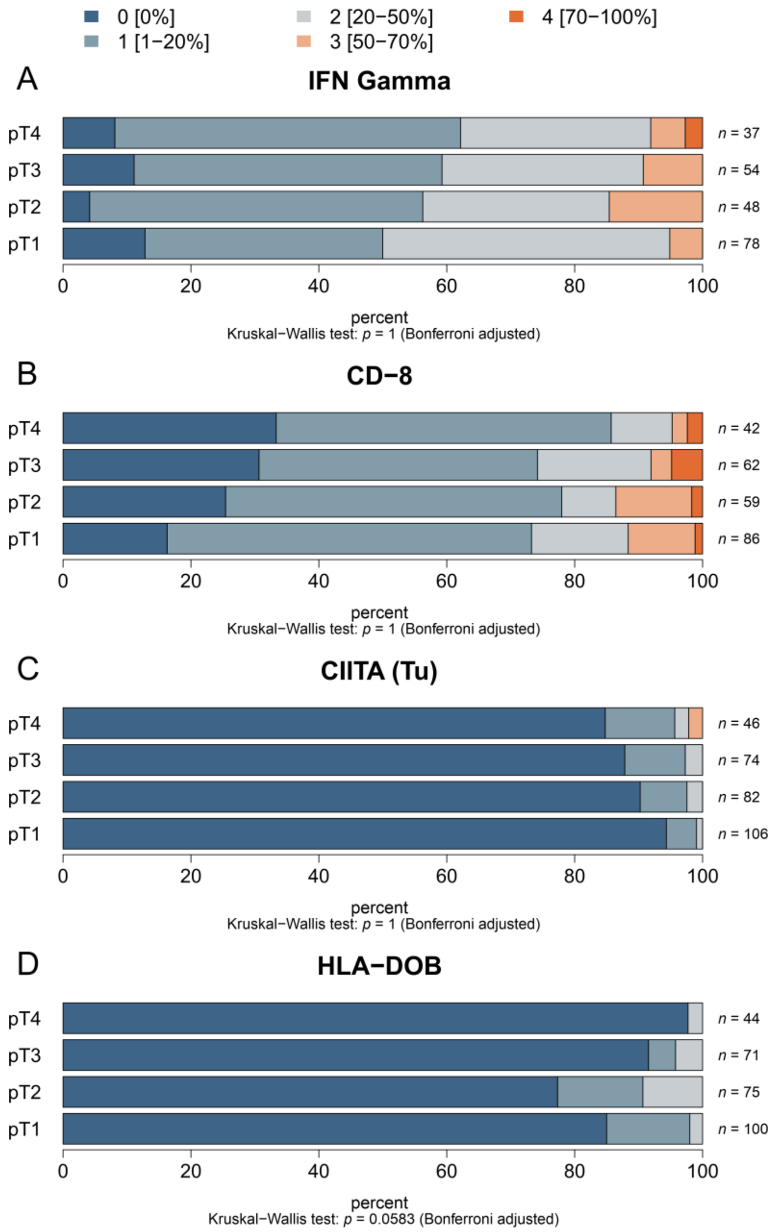

Figure S3. Correlation of the expression of (**A**) IFN-γ, (**B**) CD8, (**C**) CIITA (tu) and (**D**) HLA-DOB to pT stages of melanoma samples and/or immune cells.
